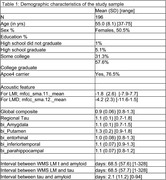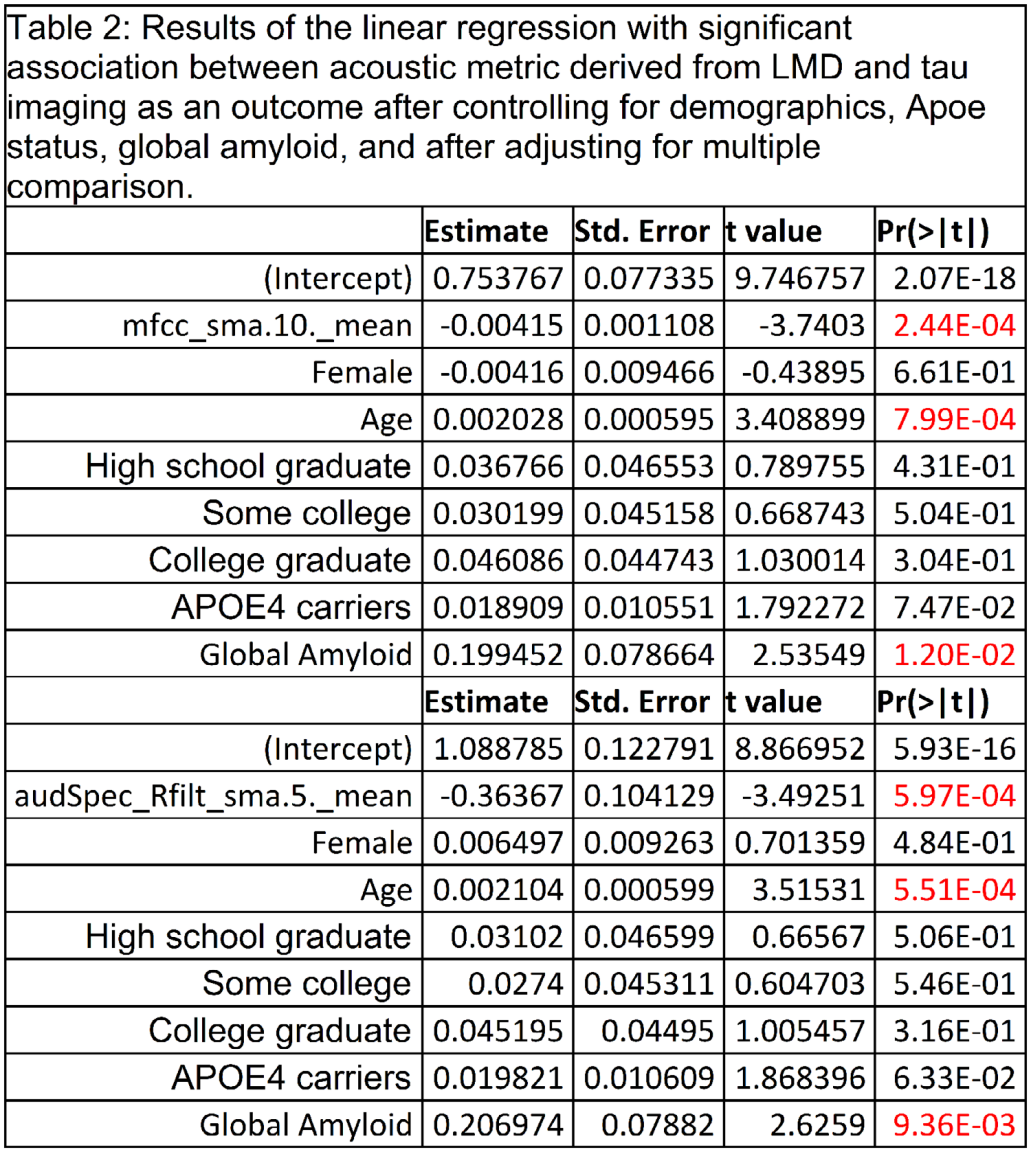# Contrasting the Association of episodic memory using traditional neuropsychological test and acoustic features with PET imaging markers

**DOI:** 10.1002/alz.091608

**Published:** 2025-01-09

**Authors:** Preeti Sunderaraman, Chenglin Lyu, Christina B. Young, Cody Karjadi, Elizabeth Mormino, Lauren Sidelinger, Sherral A. Devine, Vijaya B. Kolachalama, Honghuang Lin, Rhoda Au

**Affiliations:** ^1^ Boston University Chobanian & Avedisian School of Medicine, Boston, MA USA; ^2^ Framingham Heart Study, Boston University Chobanian & Avedisian School of Medicine, Boston, MA USA; ^3^ Boston University Alzheimer's Disease Research Center, Boston, MA USA; ^4^ Stanford University School of Medicine, Stanford, CA USA; ^5^ Department of Neurology and Neurological Sciences, Stanford University School of Medicine, Stanford, CA USA; ^6^ Framigham Heart Study ‐ Boston University, Framingham, MA USA; ^7^ Department of Anatomy & Neurobiology, Boston University Chobanian & Avedisian School of Medicine, Boston, MA USA; ^8^ University of Massachusetts Medical School, Worcester, MA USA; ^9^ Framingham Heart Study, Boston University School of Medicine, Boston, MA USA; ^10^ Department of Epidemiology, Boston University School of Public Health, Boston, MA USA; ^11^ Department of Neurology, Boston University Chobanian & Avedisian School of Medicine, Boston, MA USA; ^12^ Boston University Chobanian & Avedisian School of Medicine and School of Public Health, Boston, MA USA

## Abstract

**Background:**

Preclinical measures of Alzheimer’s disease (AD) risk include markers of amyloid and tau measured via PET scans and decline in verbal memory. Audio features from recorded speech collected during neuropsychological testing offer an alternative for monitoring preclinical AD symptoms that is nonintrusive, economical, and scalable but warrants further validation. This study aims to examine whether there is a stronger association between acoustic metrics and PET imaging biomarkers compared to a traditional verbal memory test.

**Methods:**

This study involved 196 Framingham Heart Study dementia‐free participants with available acoustic, as well as PET amyloid and tau imaging data (refer to Table 1 for demographics). For each participant, 65 acoustic features were extracted from speech samples recorded during the Wechsler's Memory Scale Logical Memory immediate (LMI) and delayed (LMD) recall tests, utilizing OpenSMILE for feature extraction. Multiple regression analyses were performed with a global composite score for PET amyloid, or PET tau for five regions (entorhinal, amygdala, parahippocampal, inferiortemporal, putamen) that were examined individually as outcomes. LMI, LMD, acoustic features derived from LMI and from LMD recorded voice segments were related to AD PET biomarkers in each regression model. Age, sex, education level, and APOE status were used as covariates for all analysis. Models using tau as outcome were adjusted for global amyloid.

**Results:**

After adjusting for multiple comparisons using FDR (significance set at 0.5), among the 65 acoustic features, the Mel‐frequency cepstral coefficient (p=0.01) and audio spectral features of the acoustic features derived from LMD (p=0.02) were most strongly associated with inferiotemporal tau region (see Table 2). No traditional measure of LMI or LMD was significantly related to any AD PET biomarker.

**Conclusions:**

In a group that was clinically asymptomatic for dementia/AD, only digital measures were associated with AD tau biomarker. These findings suggest that digital voice features have increased sensitivity to detect those at AD risk compared to traditional cognitive tests and have potential to serve as a biomarker of AD. Future studies in diverse populations are needed to test the robustness of these findings.